# Lung cancer in patients with tuberculosis

**DOI:** 10.1186/1477-7819-5-22

**Published:** 2007-02-19

**Authors:** Saulius Cicėnas, Vladislavas Vencevičius

**Affiliations:** 1 Department of Thoracic Surgery and Oncology, Institute of Oncology, Vilnius University, Santariskiu 1, Vilnius, Lithuania

## Abstract

**Background:**

Coexistent lung cancer and pulmonary tuberculosis is an urgent problem of thoracic surgery presenting a challenging task for diagnosis and surgical treatment.

**Materials and methods:**

From 1990 to 2005, 2218 patients with lung cancer underwent surgical treatment in Department of Thoracic Surgery and Oncology, Institute of Oncology, Vilnius University. In 46 (2.1%) patients coexistence of lung cancer and tuberculosis was found. Central lung cancer was diagnosed in 37 (80.4%) and peripheral – in 9 (19.6%) patients. Epidermoid cancer was diagnosed in 24 (52.2%) patients, adenocarcinoma – in 10 (21.7%) and adenoepidermoid carcinoma – in 12 (26.1%) patients. Stage I cancer was diagnosed in 12 (26.1%), stage II – in 11 (23.9%), and stage IIIA – in 23 (50%) patients.

**Results:**

Pneumonectomy was performed in 18 (39.2%), lobectomy in 10 (21.7%), bilobectomy in 10 (21.7%), segmentectomy in 8 (17.4%) patients. Postoperative surgical complications were observed in 9 (19.5%) patients, non-surgical complications occurred in 19 patients (41.3%). Six patients (13.04%) died. Combined treatment was applied to 23 (50%) patients.

**Conclusion:**

Coexistence of tuberculosis and lung cancer in thoracic surgery is fairly rare. This combination was diagnosed only in 46 cases (2.1%) out of 2218 operated lung cancer patients. Epidermoid carcinoma and stage IIIA disease was diagnosed in 50% of patients. Postoperative surgical complications occurred in 9 patients (19.5%) with lung cancer and tuberculosis. Six patients (13%) died in postoperative period. Surgery is the method of choice in treatment of combination of tuberculosis and lung cancer. Median survival of these patients was 28 ± 2 months.

## Background

Coexistence of tuberculosis and lung cancer has remained controversial since the middle of 19^th ^century. Some scientists stated that tuberculosis promotes development of cancer; others assert that tuberculosis and cancer are antagonists. Classics of pathologic anatomy P. Virchov and K. Rokitansky were advocates of latter statement (1854). As the diagnostics of lung diseases improved, scientists began to believe that post tuberculosis sclerotic changes facilitate development of cancer ("cancer in the scar") [1,2]. At present it is clear that tuberculosis and other chronic lung diseases increase the risk of lung cancer [1-4].

Well-known pathologists N. A. Dacosta and G. G. Kinare (1991) on the grounds of numerous autopsies found out that combination of lung cancer and tuberculosis of lung was found in 13.1% of patients [4]. Watanabe *et al*., published analysis of 758 of lung cancer, and coexistence of cancer and tuberculosis was found in 2.1% of cases [5,6]. Some research showed that scars, which remain after healing of tuberculoses' lesion, could cause development of lung cancer [7]. Some authors point out that increasing incidence of lung diseases is associated with increased incidence of lung cancer and therefore there should be oncological watchfulness in follow-up of patients with lung diseases or tuberculosis [8-10].

## Patients and methods

Between 1990 and 2005, 2218 patients with lung cancer underwent surgical treatment in the Department of Thoracic Surgery and Oncology, Institute of Oncology, Vilnius University. In 46 (2.1%) patients coexistence of lung cancer and tuberculosis was diagnosed. Following methods of diagnostics were used: chest X-ray, computed tomography (CT), bronchoscopy, bacterioscopic, bacteriologic and cytological investigation of sputum, pathological investigation.

Diagnosis of coexistence of cancer and tuberculosis was established before surgery in 18 (39.1%) patients in other 28 (60.8%) the diagnosis was established after pathological examination of resected specimens. Despite adequate antituberculosis treatment of all 46 pts (4–5 months of treatment with drugs of choice) in 15 (32.6%) patients mycobacterium was found in sputum before surgery (TM+).

Roentgenological, tomographical and pathological methods revealed coexistence of cancer and tuberculosis in the foci of previous tuberculosis in 32 (69.5%) patients, in 8 (17.4%) patients. changes were found unilaterally to tuberculosis, and in 6 (13.0%) changes were in the intact lung. Cases by stage are presented in Table 1.

**Table 1:** Cases with coexistent cancer and tuberculosis by stage

Stage	No of patients	%
Stage I (T1-2N0M0)	12	26.1
Stage II (T2N1M0)	11	23.1
Stage III (T3N1-2M0)	23	50

Total	46	100

Operations were performed according to criteria mentioned above and tumor extent is presented in Table 2.

**Table 2:** Operations performed in patients with coexistent lung cancer and tuberculosis

Operation	No of patients	%
Pulmonectomy	18	39.2
Lobectomy	10	21.7
Bilobectomy	10	21.7
Segmentectomy	8	17.4

Total	46	100

It should be noted that such traumatic operations were performed because of clinical situation. Pleural adhesions, fibrosis and sclerosis of lung tissue and scarry changes are usually observed in tuberculosis patients.

Pathistological investigation of removed specimen revealed that fibrocavernous tuberculosis in 15 (30.5%) patients, infiltrative in 12 (26.0%), disseminated in 5 (10.8%) and pneumosclerosis due to tuberculosis (large calcinates, hard foci, solitary and multiple tuberculomas, lung cirrhosis) in 14 (30.6%) patients respectively. Morphological type of cancer is presented in Table 3.

**Table 3:** Morphology of cancer in operated patients

Morphology of tumor	No of patients	%
Epidermoid carcinoma	24	52.2
Adenocarcinoma	10	21.7
Epidermoid adenocarcinoma	12	26.1

Total	46	100

In 12 (26.0%) patients with progressive lung cancer that was located in the region of post tuberculosis changes we observed destruction of tumor with destruction of lung tissue and tuberculosis dissemination around the tumor. Adjuvant treatment was given to 23 (50%) patients: Of these, 12 (52%) received radiation therapy (42 Gy), 11 (48%) received chemotherapy (etoposide+cisplatinum × 6 cycles), 5 (21.7%) patients from this group later received antituberculosis treatment because of exacerbation of tuberculosis.

## Results

Type and extent of operation depended on general status of patient, concomitant disease, extent of cancer and tuberculosis. Clinical type and phase of tuberculosis as well as anatomical, tomographical and histological type of lung cancer were established by roentgenological and morphological investigations.

**Figure 1:**
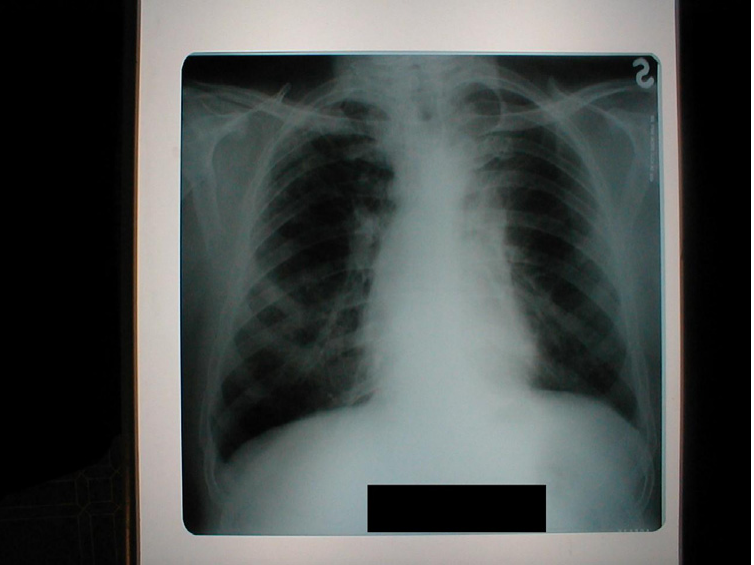
**X-ray of the thorax**. Infiltrative changes are observed in the left upper lobe.

**Figure 2:**
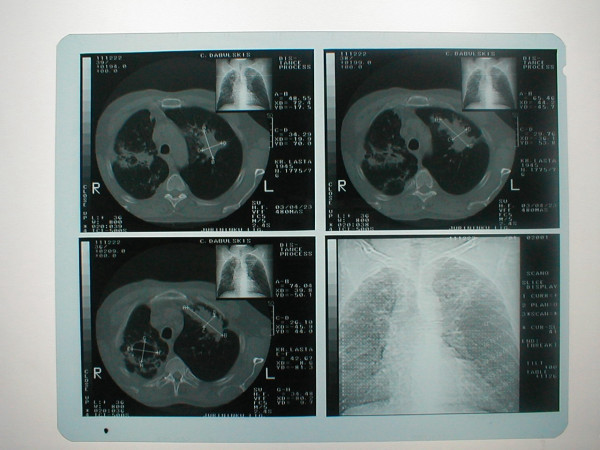
**Chest CT**. There is pulmonary bulla in the right S1 segment, and focus of mottled opacity 7,5 × 2,5 cm with infiltration around it.

**Figure 3:**
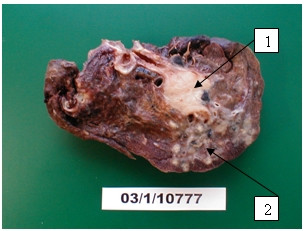
**Postoperative specimen**. Tumor occludes lumen of bronchus (1) and tuberculosis(2).

**Figure 4:**
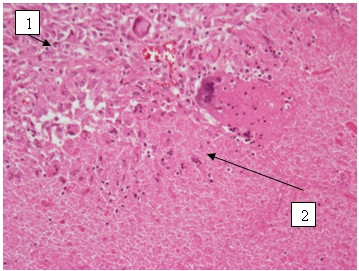
**Microview of resected specimen H&E stain, ×20**. Tumor cells (1) and tuberculosis changes (2) are seen.

For example this 58-year-old man with the history of chronic bronchitis for about 30 years of duration was diagnosed with tuberculosis 5 months ago. Treatment with five basic antituberculosis medications was started. Treatment was declared ineffective after the bacteriological and bacterioscopical investigations revealed tuberculosis mycobacterium in sputum (TM+). Infiltrative changes in the upper lobe of left lung were found at x-ray examination (Figure 1). Figure 2 shows chest CT scan with pulmonary bullous in right upper lobe. Brush-biopsy was performed during fiber optic bronchoscopy, and cells of epidermoid cancer were found. Patient underwent surgery. Left upper lobectomy was performed. Pathological investigation revealed infiltrating epidermoid carcinoma of lung (pT1N0M0). Figure 3 shows macroview of postoperative specimen. Because of radical surgery no anticancer treatment was prescribed. Treatment with antituberculosis medications was performed. Figure 4 shows morphological changes in resected specimen.

Microscopy of the resected specimen showed cancer cells against the background of tuberculosis changes. This specimen confirmed coexistence of tuberculosis and lung cancer, and in this case one process stimulates another. This could explain ineffectiveness of antituberculosis treatment. Exacerbation of tuberculosis influenced incidence of postoperative complications.

Postoperative complications occurred in 28 (60.8%) of our patients. From these 9 (19.5%) were surgical complications (Table 4). Rest 19 (41.3%) complications were associated with chronic pneumonia (3 patients), bronchial asthma (3 patients), COPD (9 patients) and heart failure (4 patients).

**Table 4:** Postoperative surgical complications

Postoperative surgical complications	No. of patients	%
Insufficiency of suture of bronchial stump	3	6.5
Subcutaneous emphysema	4	8.7
Postoperative wound infection	2	4.3

Total	9	19.5

Six patients (13.4%) died due to postoperative complications. Postoperative radiotherapy or chemoradiotherapy was applied in 23 (50%) patients with inactive tuberculosis. From these 12 (52%) patients with pT3N1M0 were treated with regional radiotherapy. The other 11 (48%) patients with T3N2M0 were treated with regional radiotherapy and chemotherapy (6 courses of etoposide + cisplatinum). Tuberculosis specialists and oncologists conducted further follow-up. Median survival of these patients was 28 ± 2 months.

## Discussion

Diagnosis of lung cancer in patients with tuberculosis or with residual effects of tuberculosis possesses some peculiarities. These depend on variety of clinical symptoms that occurs due to coexistence of diseases (which is called mix), clinical course and site of cancer (both processes are located in the same region or separately). When cancer develops against the background of active tuberculosis, common symptoms are worsening of patients' general status (fever, signs of intoxication, shortness of breath and often sputum with touch of blood occur). Investigations of show that x-ray picture in these cases is different: foci of infiltration of various size, atelectasis, hypoventilation of various parts of lungs, and foci of destruction with formation of caverns is seen. Bacteriological analysis shows presence of tuberculosis mycobacterium, which was not previously detected. As shown earlier in cases, when new signs not typical for tuberculosis occur there should be dynamic analysis of roentgenological signs and bacteriological analysis. In case of ineffective specific treatment coexistence of lung cancer and tuberculosis should be presumed [2].

According to Drent (1994), Tamura (1999), and Watanabe (1999) post tuberculosis scars deform blood and lymphatic vessels [3,5,6]. Lymphostasis, conditions for deposit of carcinogens and development of malignant process occur. In patients with coexistence of tuberculosis and lung cancer diagnosis is difficult. In such cases bronchoscopy, CT and transthoracic lung biopsy should be performed. If diagnosis is unclear after all performed diagnostic tests, operation is necessary.

In our series of 2218 operated patients, 46 (2.1%) cases of coexistence of lung cancer and tuberculosis were found. It is possible that tuberculosis of lung prepare conditions for the development of lung cancer. We think that patients with coexistence of lung cancer and tuberculosis could be divided into three groups:

1. Tuberculosis and lung cancer are unrelated

2. There is relation between both processes, post tuberculosis changes, deformation of bronchi and alveoli, epithelial dysplasia are risk factors for lung cancer.

3. In lung cancer progression old foci of tuberculosis reactivate and dissemination of tuberculosis mycobacterium occur.

We agree with scientists who affirm that in case of new lung symptoms patients with tuberculosis after recovery should undergo follow-up periodically. In order to diagnose lung cancer early, chest x-ray, bronchoscopy and cytological investigation of sputum should be performed. It should be noted that mycobacterium was found in sputum again in part of patients with history of tuberculosis. It was thought to be recurrence of tuberculosis and these patients received recurring antituberculosis treatment. However, when careful checkup was performed in cases of worsening general status in patients with history of tuberculosis, usually lung cancer was diagnosed. Therefore periodic checkup of these patients is necessary.

## Conclusion

Coexistence of tuberculosis and lung cancer is obvious, although it is rare in the practice of thoracic surgery. In most cases epidermoid cancer was diagnosed (52.2% of patients). Stage IIIA lung cancer was diagnosed in 50% of patients. Postoperative surgical complications occurred for 9 (19.5%) patients. 19 (41.35%) non-surgical complications occurred due to heart and lungs chronic diseases. Six patients (13.04%) died. Surgery is treatment of choice in cases of coexistence of tuberculosis and lung cancer. A good median survival is normally achievable.

## Conflict of interests

The author(s) declare that they have no competing interests.

## Authors' contributions

SC – collected number of patients, operated and treated them, used diagnostic methods.

VV – wrote manuscript, send speciments to pathologist, prepared material for publications and operated patients.
